# The role of Visual Evoked Potential (VEP) in monitoring the progression and in guiding the treatment of glaucoma patients with poor compliance

**Published:** 2020

**Authors:** Anne Marie Firan, Raluca Claudia Iancu, Inna Adriana Bujor, Radu Constantin Ciuluvică, Ruxandra Tudosescu, Emil Ungureanu, Irena Gabriela Pașca, Liliana Voinea, Sînziana Istrate

**Affiliations:** * Barnsley District Hospital, NHS trust, England; ** Department of Ophthalmology, University Emergency Hospital, Bucharest, Romania; *** “Carol Davila” University of Medicine and Pharmacy, Bucharest, Romania; **** Department of Ophthalmology, “Regina Maria” Private Health Care, Bucharest, Romania

**Keywords:** visual evoked potential, glaucoma, amplitude, latency

## Abstract

**Objectives:** The objectives of the present study were to analyze the possibility of using pattern VEPs (VEP of pattern type) in glaucoma patients and their role in the follow-up and coordination/ management of anti-glaucoma treatment.

**Patients and Methods:** This is a prospective study on 54 eyes of 30 consecutive glaucoma patients, whose compliance capacity did not allow automatic perimetry and OCT scan to be carried out.

The patients were divided into two groups: group A – the study group and group B – the control group.

All patients underwent FO exam and pachymetry, plus VEP exam for group A patients.

Patients were analyzed at the initial visit and at 1 month, 3, 6, 12 months.

Statistical analysis was made using t-test, ANOVA, Fisher test and Pearson correlation coefficient.

**Results:** These participants presented a positive correlation between C/ D ratio and the latency of the P100 wave at 1 degree and a negative correlation between C/ D ratio and the amplitude of the P100 wave both at 1 degree (60 arc minutes) and at 0.25 degrees (15 arc minutes). During the study, the values of the latent P100 changed statistically at 6 months and at 1 year.

Using all the data, the authors of the study considered it necessary to modify the treatment for 2 patients out of 13 in group B and for 9 patients out of 16 in group A (p = 0,02892).

**Conclusions:** VEP supplies additional/ further data that significantly help guiding the treatment and monitoring the progression, therefore, it should be part of the routine examination for these patients.

Further studies are necessary to deepen our understanding of the visual evoked potentials utility.

## Introduction

Glaucoma is the most important cause of permanent vision loss and, once diagnosed, glaucoma patients need to be frequently and continuously monitored through their lives, and their treatment should be adjusted considering the permanent changing parameter, in order to preserve a satisfying visual function.

Visual evoked potentials are represented by the measurement of an electric impulse at the occipital level following a light stimulation. As the cortical projection area of the fovea and macula in generalis much larger than that of the peripheral retina, VEP mainly has in view signs with central origin and peripheral defects that may not be rendered evident by VEP analysis. Of the VEP analyses available in retinal abnormalities, a special interest was shown to PRVEP (Pattern Reversal Visual Evoked Potential), which represents an initial negative peak at about 70 ms (N70), then a positive peak (P100) at about 100 ms, followed by another negative peak at about 155 ms (N155). These can be used by stimulation on 15 arcminutes (0.25 degrees) or by stimulation on 60 arcminutes (1 degree). VEPs proved to be effective in detecting and monitoring glaucomatous progress [**1**-**4**].

To our knowledge, no results of the VEPs measurements have been able to replace automatic perimetry and/ or optical coherence tomography examination (OCT) of the optic nerve, even if there were studies that promisingly correlated with the perimetry modifications or OCT scan of the optic nerve.

In spite of all advances of perimetry and OCT, there are still patients for whom those examinations cannot be carried out with satisfying precision due to compliance difficulties, i.e. elderly patients exhibiting concentration issues as well as patients of any age with attention deficit or with motor of psychic dysfunctions. 

## Aim of the study

The aim of the study was to analyze the possibilities of using pattern type VEPs in glaucoma patients and their role in the follow up and management of glaucoma treatment.

## Patients and Methods

This is a prospective study on 30 consecutive glaucoma patients, whose compliance made impossible to perform all glaucoma specific investigations, and specifically, patients in whom automatic perimetry and OCT scan of the optic nerve could not be performed in order to determine the progression of the glaucoma. The University Emergency Hospital Ethics Committee approval was obtained and an Informed Consent about the study procedures and visits schedule was signed by all participants.

These patients were divided into two groups:

- The study group included 16 patients for whom visual acuity exam, Goldman applanation tonometry, biomicroscopic exam of fundus oculi (FO) with 90 diopter lens and Visual evoked potentials of pattern type (flash and PRVEP = pattern reversal visual evoked potential), were carried out;

- The control group included 16 patients for whom visual acuity examination, Goldman applanation tonometry, FO, biomicroscopic exam with the 90-diopter lens, were carried out.

***Inclusion criteria:***

- Patients over 18 years of age diagnosed with primary glaucoma with open angle, with or without treatment.

- Patients in whom reduced compliance did not allow automatic perimetry and OCT scan.

- Patients in whom visual acuity could be measured.

- Patients from whom informed consent could be obtained.

- Patients with iridocorneal open angle at indirect gonioscopic exam (Goldmann lens): III or IV grade angle on at least two quadrants, were included.

***Exclusion criteria:***

- Patients with significant transparency modifications of the transparent media.

- Patients with refraction error with the sphere over +/ - 5 diopters or astigmatism greater than +/ - 2 diopters.

- Patients with concomitant vascular retinal disease.

- Patients with edema of the optic nerve; either stasis edema or associated to an ischemic neuropathy.

- Patients with a history of intraocular injections with corticosteroids.

- Patients with a history of intraocular injections with vascular endothelial growth factor (VEGF) inhibitors on the respective eye.

- Amblyopia.

- Patients who have presented cerebral vascular strokes or transitory ischemic strokes.

- Patients with inflammatory lesions of neuritis type, irrespective of their location.

- Patients who underwent eye surgery in the past 6 months, exception: 3 months previous non-complicated surgery for cataract.

- Patients with significant macular abnormalities.

Study group A (**Table 1**) was represented by 16 patients (27 eyes). Investigations were represented by visual acuity, applanation tonometry, gonioscopy, fundus examination with the 90-diopter lens, retinophotography – if possible – and the pattern visual evoked potentials (PRVEP and flash) with stimulation on 1 degree (60 arc seconds) or on 0.25 degrees (15 arc seconds).

**Table 1:** Study group A results at the initial visit

Name	Sex	Age	VA	IOP	Gonio	Pachy	c/d ratio	Pat lat 1gr	Pattern amp 1gr	Patlat 15	Pattern 15 amp	Fla-ampl	Fla lat
AE	F	83	0.9	18	3	518	0.5	113.3	11.6	143	14.9	3.76	108
AE	F	83	0.8	20	3	520	0.9	148.5	3.98	120.9	2.6	5.3	103.3
BM	F	65	0.5	21	4	549	0.3	108	9.07	129.2	12.3	7.02	148.4
BM	F	65	1	22	4	536	0.5	109.2	12	125	9.05	4.87	134.3
BN	M	58	1	16	2	530	0.7	115.1	7.88	126.2	8.75	8.21	113.7
BN	M	58	1	18	2	528	0.6	116	7.71	120.9	9.89	5.48	112.7
BG	F	72	1	16	3	538	0.5	128.6	7.33	142.7	9.2	16.8	129.6
BT	F	59	1	15	3	542	0.5	122.7	11.1	140.3	9.19	19.1	133.4
CB	M	81	0.9	20	4	550	0.7	113.9	6.14	129.7	4.39	4	99.6
CB	M	91	0.8	20	4	555	0.7	120.4	6.34	131.5	2.91	1.62	98.6
DI	F	76	0.9	18	4	532	0.4	110.4	8.34	128	17.4	11.7	111.8
DI	F	76	0.9	21	4	530	0.5	109.8	11.4	123.3	18.8	9.58	107.1
DA	F	75	0.6	23	2	574	0.8	132.1	5	118	1.73	14.1	157.7
DA	F	75	0.6	19	3	580	0.8	145	2.73	112.7	2.82	1.53	118.1
EA	M	67	1	23	3	520	0.75	118.6	12.5	134.4	18.4	0.46	133.4
EA	M	67	1	19	3	518	0.7	116.8	14.5	123.9	21.3	7.96	132.4
FI	F	83	0.8	22	3	538	0.75	102.7	11.2	125	11.2	8.47	84.5
FI	F	83	1	24	2	550	0.3	104.5	12.1	122.7	13.2	10.5	85.5
GFG	F	66	0.3	35	3	550	0.6	128	0.29	76.9	1.21	3.82	107.1
GFG	F	66	0.5	19	3	525	0.85	116.8	2.33	76.3	1.41	0.63	112.7
GE	M	81	0.9	21	3	533	0.5	116.8	5.04	132.1	11	3.45	131.5
GE	M	81	0.9	22	3	532	0.8	113.3	5.17	127.4	12.3	1.8	134.3
GF	F	76	0.4	35	2	550	0.6	128	0.29	76.9	1.21	3.82	107.1
GF	F	76	0.5	19	3	525	0.85	116.8	2.33	76.3	1.41	0.63	112.7
IA	F	58	0.9	28	3	560	0.65	113.3	12.3	135.6	8.49	10.2	116.5
IM	F	82	0.9	20	3	541	0.6	109.8	12.6	118.6	11.7	12.5	113.7
LF	M	81	1	14	4	520	0.4	115.7	10.9	128	26.3	13.8	123.1

Group B, the control group, was represented by 14 patients (27 eyes). Investigations were similar except for PRVEP and flash. During the study, one patient missed part of the study visits and was removed from the group; thus, only 13 patients (25 eyes) remained in this group (**Table 2**).

**Table 2:** Control group B results at the initial visit

Name	Sex	Age	VA	IOP	Gonio	Pachy	C/ D ratio
LM	M	83	0.9	20	grade 3	524	0.65
LM	M	83	0.9	19	grade 3	526	0.7
NE	F	73	0.9	18	grade 3	524	0.7
NE	F	73	0.9	19	grade 3	526	0.7
OI	M	80	0.4	26	grade 4	530	0.8
OI	M	80	1	21	grade 4	528	0.4
PA	F	64	0.3	20	grade 4	528	0.7
PA	F	64	0.5	22	grade 4	530	0.8
PG	F	81	0.9	19	grade 3	535	0.7
PG	F	81	0.9	20	grade 3	537	0.7
PI	M	65	0.7	12	grade 3	543	0.9
PI	M	65	0.8	12	grade 3	538	0.85
PS	F	83	0.8	19	grade 3	530	0.9
PS	F	83	0.8	20	grade 3	524	0.65
PT	F	58	1	23	grade 3	545	0.5
PT	F	58	0.8	22	grade 4	522	0.8
RA	M	65	0.9	21	grade 3	556	0.2
RA	M	65	0.9	20	grade 3	560	0.2
RD	F	76	0.8	20	grade 2	555	0.2
RD	F	76	1	19	grade 3	549	0.4
RML	M	81	1	17	grade 2-3	527	0.7
RML	M	81	1	17	grade 2-3	533	0.85
SC	M	59	1	18	grade 2	512	0.4
TE	F	72	1	15	grade 3	530	0.35
TE	F	72	1	15	grade 3	526	0.35

The patients were followed up for 5 visits – the first, then at 1, 3, 6 months and 1 year. Slit lamp exam, visual acuity exam, fundus exam and IOP measurement were carried out at each visit.

## Results

**Fig. 1:**
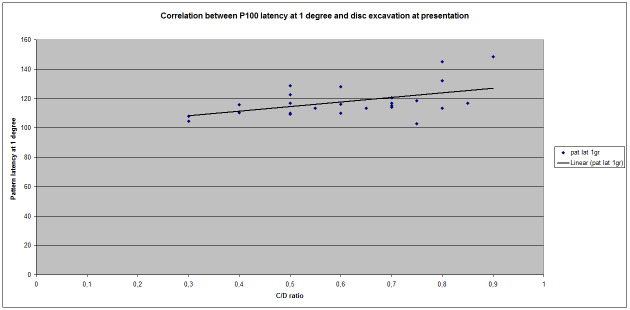
Correlation between P100 latency at 1 degree (60 arc minutes) and disc excavation at presentation

A direct correlation between VEP latency at 1 degree and C/ D ratio of the optic disk evaluated by FO exam was observed at the initial visit (V0) for group A (**Fig. 1**).

**Fig. 2:**
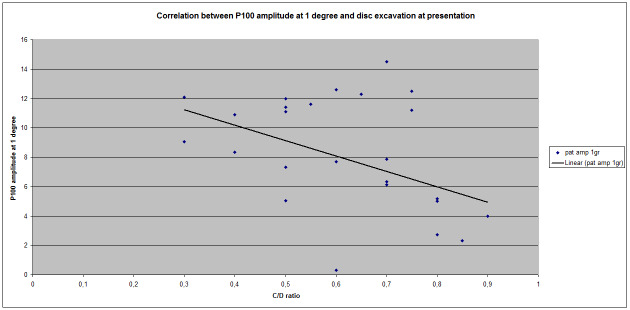
Correlation between P100 amplitude at 1 degree (60 arc minutes) and disc excavation at presentation

The Pearson index for this correlation was 0,469893, which indicated a positive correlation of medium value. Regarding the correlation between P100 amplitude at 1 degree and the size of the excavation, a negative correlation (Pearson index = - 0,421838318) not as strong as latency correlation was observed (**Fig. 2**).

**Fig. 3:**
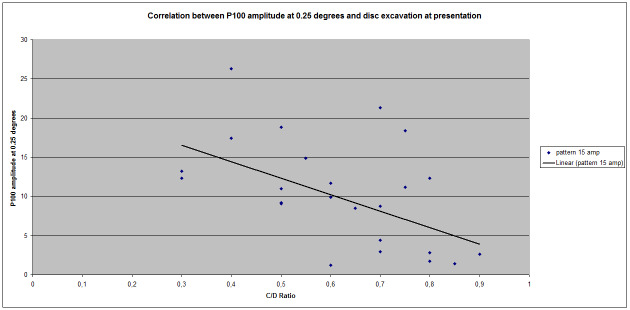
Correlation between P100 amplitude at 0.25 degrees and disc excavation at presentation

No significant correlation between P100 latency at 0.25 degrees (Pearson index = 0,283411325) and C/ D ratio was obtained, but we have noticed a significant negative correlation between excavation and the P100 amplitude at 0,25 degrees (Pearson index = -0,516267568) (**Fig. 3**).

These strong correlations between C/ D ratio and amplitude might be explained by the group composition, which had a relatively larger proportion of advanced glaucoma as compared to global glaucoma population.

At subsequent visits, C/ D ratio in group A remained identical, except for 3 patients: the excavation of LE in patient B.M. increased on LE from 0,5 to 0,6; in patient F.I. left eye C/ D ratio increased from 0,3 to 0,5 and in patient G.F. increased both on RE (from 0,4 to 0,5) and on LE (from 0,5 to 0,6). On the other hand, VEP modifications were significant for most patients, even without changes in fundus exam (**Table 3**).

**Table 3:** P100 latency evolution between visits in group A

Initial	1 month	3 months	6 months	1 year
113,3	112,8	114,8	113,9	115,3
148,5	149,3	148,7	151,2	150,1
108	108,6	108,2	107,6	107,8
109,2	109,5	109,7	109,3	110,6
115,1	115,4	115,2	116,4	118,1
116	114,5	114,7	112,9	113,6
128,6	132,4	135,7	130,3	133,6
122,7	120,8	124,8	126,7	125,1
113,9	114,7	118,4	120,6	118,7
120,4	120,8	120,7	128,4	129,2
110,4	114,2	107,3	111,6	112,4
109,8	111,4	113,5	111,7	115,7
132,1	135,7	136,6	138,9	137,5
145	149,8	146,2	146,4	145,9
118,6	121,5	126,8	130,2	130,5
116,8	116,4	123,7	127,9	125,7
102,7	111	114,8	115,6	114,6
104,5	101	102,8	103,5	102,9
128	133,1	131,9	135,8	136,3
116,8	118,2	119,5	120,6	123,7
116,8	115,7	117,4	119,9	121
113,3	116,4	119,4	122,3	125,7
128	128,8	130,6	132,4	133,5
116,8	117,4	119,5	122,3	121,8
113,3	114,1	113,6	117,4	116,9
109,8	114,2	116,5	120,3	119,4
115,7	116,4	115,2	115,9	117,1

Thus, at the initial visit, the medium value of the P100 latency at 1 degree was 118.3 (+/ - 4,3211 for 95% CI; +/ - 5,8313 for 99% CI). At the final visit, the medium value was 123,063 (+/ - 4,4584 for 95% CI; +/ - 6,0167 for 99% CI). Between the first and final visit there was a statistically significant difference (p 0,0001) for P100 latency at 1 degree (**Table 4**). 

**Table 4:** Analysis of statistical significance of P100 latency between initial visit and final visit

*Data Summary*.					
	A	B	Total		
n	27	27	54		
∑ X	3194.100000	3322.700000	6516.800000		
∑ X2	380950.79	412189.5299	793140.3199		
SS	3086.76	3288.223	6683.2415		
mean	118.3	123.063	120.6815		
*Results*					
Mean a – Mean b	t	df	p	one-tailed	<.0001
-4.763	-6.11	26		two-tailed	<.0001

At the 6 months visit, the medium value of P100 latency at 1 degree was 122,5926 (+/ - 5,8313 for 99% CI, +/ - 6,1829 for 99% CI), which has been significant (p<0,0001). At the 3 months visit, the medium value of the P100 latency at 1 degree was 120,9704 (+/ - 4,4532 for 95% CI, +/ - 6,0096 for 99% CI), not statistically significant (p = 0,377565) (**Table 5**).

**Table 5:** Analysis of statistical significance of P100 latency between initial visit and at 3 months visit

*Data Summary*.					
	A	B	Total		
n	27	27	54		
∑ X	3194.100000	3266.200000	6460.300000		
∑ X2	380950.79	398393.9599	779344.75		
SS	3086.76	3280.5363	6465.5631		
mean	118.3	123.063	120.6815		
*Results*					
Mean a – Mean b	t	df	p	one-tailed	0.1887825
-2.6704	-0.89	52		two-tailed	0.377565

At the 1-month visit, the medium value of P100 latency at 1 degree was 119,7815 (+/ - 4.5701 for 95% CI, +/ - 6,1674 for 99% CI), also not statistically significant (p = 0.626195).

In group B, the modification of C/ D ratio was noticed in 3 patients. In patient T.E., it changed both for RE (from 0,35 to 0,5) and for LE (from 0,35 to 0,4). In patient P.A., the C/ D ratio increased on RE from 0,75 to 0,8. In patient R.A. it increased on RE from 0,2 to 0,4. 

During the study, it was necessary to modify the anti-glaucomatous treatment for 2 patients out of 13 in group B. For group A, change of treatment has been necessary for 9 patients out of 16 (Fischer test p = 0.02829688993848931). Therefore, supplementary data helped adjust the treatment for more patients in group A as compared to group B, and the difference was significant from a statistical point of view. 

## Discussions

Examination in glaucoma includes certain mandatory exams, such as applanation tonometry, fundus exam and automatic perimetry. Besides these, there are other types of examinations that can be supplementary or complementary. The choice of tests to be used should be considered according to how much information they are able to provide and according to how easily they can be carried out for the respective patient.

In order to determine how informative such a test is, we have to consider its prediction power, its sensitivity and specificity, as well as its relevance and variability. Other types of information may refer to costs and time necessary to carry it out.

PRVEP was a useful tool in glaucoma diagnosis and progression monitoring when compared to the visual field (MD = mean defect and PD = pattern deviation), with different results between studies. Thus, Mokbel TH and Ghanem AA and, respectively, Cothary et al. obtained a negative correlation between MD and the P100 latency, while Grippo et al. did not obtain a clear correlation between MD and the P100 latency. To our knowledge, no correlation between PRVEP and RNFL or various sectors of RNFL has been analyzed in these patients.

There are studies that analyzed the way the patients perceived these investigations. In addition, Gardiner et al. [**5**] followed up 7 tests that the patients had to place in order between 1 (the most comfortable) and 7 (the least comfortable). In this study, the patients indicated Goldmann applanation tonometry on the first place (median/ average place 2.5), followed by HRT (median place 3.3), double frequency perimetry and VEP (median place 4), then automatic perimetry (median place 4.8) and SWAP automatic perimetry (median place 5.3). Bjerre et al. also showed that the patients preferred VEP in comparison with automatic perimetry SITA [**6**].

Unlike other studies, the present prospective study included patients whose compliance capacity did not allow automatic perimetry and OCT exam to be carried out. The study analyzed if, under these circumstances, VEP examination could be performed within the routine management of these patients.

## Conclusions

- To our knowledge, no results of VEP measurements, able to replace automatic perimetry and/ or OCT exam of the optic nerve, have been determined so far, even if there have been studies in which OCT modifications correlated to the perimetry modifications or those of the OCT scan of the optic nerve;

- There are glaucoma patients who, due to varied reasons, are not compliant enough to be able to carry out these tests.

- These patients are usually older persons, as age also represents one of the three significant risk factors for the development of glaucoma; these patients will frequently develop more advanced glaucoma than normal glaucomatous population.

- Even as early as the first visit, these patients presented a positive correlation between the C/ D ratio and the P100 latency at 1 degree and a negative correlation between C/ D ratio and the P100 amplitude both at 1 degree (60 arcminutes) and at 0.25 degrees (15 arcminutes);

- For these patients, the C/ D ratio variation in this study was not significant, but the P100 latency has varied significantly at 6 months and 1 year compared to the initial visit. For 1 month and 3 months visits, the difference was not significant compared to the initial visit;

- The data thus obtained led to the modification of treatment in 2 patients out of 13 for group B and in 9 patients out of 16 in group A (Fisher test, p = 0.02892698993848931);

- The authors of the study consider that VEP provides supplementary data that significantly helps guide treatment and monitor progression.

- For these patients, we recommend the PRVEP evaluation at a 6 months interval;

- We intend to follow up these patients for a longer period and add new patients, in order to deepen our understanding of the visual evoked potentials utility.

**Acknowledgements**

The authors gratefully acknowledge Programul Operațional Capital Uman 2014–2020 for the data provided by Heidelberg HRT III supported through POCU/91/4/8/109169 (Cod SMIS 2014+: 109169) “Diagnosticul si terapia bolilor rare sistemice cu afectare oculara - OCURARE. All authors have equal contribution to the paper. 
